# RWR-algorithm-based dissection of microRNA-506-3p and microRNA-140-5p as radiosensitive biomarkers in colorectal cancer

**DOI:** 10.18632/aging.103907

**Published:** 2020-10-08

**Authors:** Fei Liao, Xin Chen, Pailan Peng, Weiguo Dong

**Affiliations:** 1 Department of Gastroenterology, Renmin Hospital of Wuhan University, Wuhan 430060, China; 2 Department of Oncology, Renmin Hospital of Wuhan University, Wuhan 430060, China; 3 Department of Gastroenterology, The Affiliated Hospital of Guizhou Medical University, Guiyang 550004, China

**Keywords:** colorectal cancer, radiosensitivity, microRNA, random walk with restart

## Abstract

Radiotherapy resistance is one of the main causes for treatment failure in colorectal cancer (CRC), and it is associated with the deregulation of certain microRNAs. In this study, we constructed the microRNA-mRNA network consisting of 2275 microRNAs and 7045 target genes, collected the known microRNAs related to CRC-radiosensitivity (CRCR) (n=18) as the seed nodes, and applied the algorithm of random walk with restart (RWR) to the network to identify novel CRCR-related microRNAs (n=263). In functional analysis, 263 novel microRNAs shared a high proportion of the same biological processes and pathways with the known microRNAs. In topological analysis of the sub-network of the 263 microRNAs and their targets, hsa-mir-506-3p and hsa-mir-140-5p were identified as network hub nodes. In plasma, radiosensitive patients had a higher expression level of hsa-mir-506-3p and hsa-mir-140-5p than radioresistant patients. In experimental validation, both hsa-mir-506-3p and hsa-mir-140-5p over-expression could obviously decrease the cell proliferation, survival rate and colonality in CRC cells after radiation. In conclusion, this study combined the novel network-based method with experimental validation, and identified two novel radiosensitive biomarkers of hsa-mir-506-3p and hsa-mir-140-5p in CRC.

## INTRODUCTION

Colorectal cancer (CRC) is one of the most common cancers worldwide, with an estimated 1.4 million new cases and 693,900 deaths per year [1]. Currently, surgical resection remains the only curative treatment for CRC. However, about 20% to 40% CRC patients are initially diagnosed at a locally advanced, unresectable, nonmetastatic stage termed “locally advanced CRC” [2]. Neoadjuvant chemoradiation is the standard therapeutic strategy for these patients. Unfortunately, many CRC patients are radioresistant and have a poor prognosis [3]. Therefore, it is necessary to elucidate the underlying radioresistant mechanisms in CRC, which may ultimately improve the therapeutic outcomes.

MicroRNAs are a class of small non-coding RNAs containing about 18~24 nucleotides. MicoRNAs function as post-transcriptional regulators, and usually cause translational suppression or RNA degradation via specific binding to 3′-untranslated region (3′-UTR) of their target genes [4]. Accumulating evidence has suggested a strong association between microRNAs deregulation and tumor radioresistance. For example, microRNA-21 expression could promote the radioresistance in a variety of cancer cell lines, including breast, lung, glioblastoma and nasopharyngeal cancers [3, 5–8]. Several microRNAs were also found dysregulated in radioresistant CRC cell lines, and upregulation or downregulation of these microRNAs could protect the cells from radiation effects. However, more novel microRNAs biomarkers are needed to illuminate the underlying mechanism of CRC radioresistance.

In recent years, studies have investigated the mechanism of several diseases with the help of advanced computational methods, such as the network-based method. Some studies adopted the gene-gene interaction network, which allowed us to identify novel disease genes based on known disease genes [9]. In this study, we adopted a classic network-based algorithm of random walk with restart (RWR), and developed a three-stage filtration strategy to identify novel CRC-radiosensitivity (CRCR)-related microRNAs based on a microRNA-mRNA network and known CRCR-related microRNAs. For the network hub microRNAs, we also conducted an experimental validation.

## RESULTS

### CRCR-related microRNAs identified by the RWR algorithm

**Figure 1:**
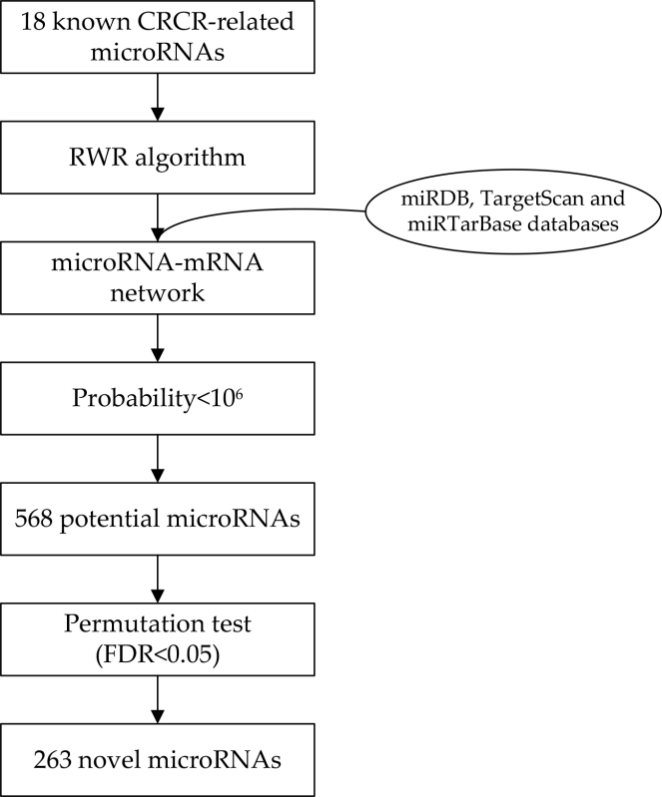
**Flowchart of the network-based method to identify radiosensitivity-related microRNAs in colorectal cancer.**

**Figure 2:**
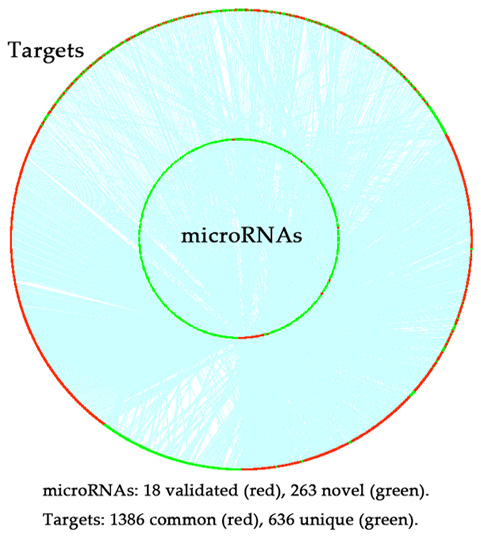
**Distribution of the targets of 18 experimentally validated microRNAs and 263 novel microRNAs predicted by the random walk with restart (RWR) algorithm.** Red dot in the inner ring, experimentally validated microRNAs; green dot in the inner ring, novel microRNAs; red dot in the outer ring, common targets of the experimentally validated microRNAs and novel microRNAs; green dot in the outer ring, unique targets of the experimentally validated microRNAs or novel microRNAs; blue line, the interaction between microRNAs and the targets.

Eighteen microRNAs were collected by literature review, which has been experimentally validated in association with CRCR (Table 1). By integrating three microRNA databases, a microRNA-mRNA network was constructed consisting of 2275 microRNAs and 7045 target genes. Then, we selecting those 18 microRNAs as seed nodes, and conducted the RWR algorithm analysis on the network (Figure 1). After permutation test, 263 novel CRCR-related microRNAs were identified (Supplementary Table 1). There were 1386 common targets between the 18 microRNAs and 263 microRNAs (Figure 2).

**Table 1:** Characteristics of radiosensitivity-related microRNAs collected from literature in colorectal cancer.

**Reported ID**	**Official ID**	**Year**	**Target**	**Radiosensitivity**	**PMID**
let-7e	hsa-let-7e-5p	2018	IGF-1R	Increased	30515804
miR-185	hsa-mir-185-5p	2018	IGF1R, IGF2	Increased	29990869
miR-369-3p	hsa-mir-369-3p	2018	DYRK1A	Decreased	29773344
miR-519b-3p	hsa-mir-519b-3p	2018	ARID4B	Increased	29459645
miR-214	hsa-mir-214-3p	2018	ATG12	Increased	29459645
miR-155	hsa-mir-155-5p	2017	PTEN	Decreased	28879560
miR-222	hsa-mir-222-3p	2017	FOXO3a	Decreased	28879560
miR-195	hsa-mir-195-5p	2017	CARM1	Increased	28255246
miR-145	hsa-mir-145-5p	2017	-	Decreased	27696511
miR-29a	hsa-mir-29a-3p	2016	PTEN	Decreased	27548517
miR-630	hsa-mir-630	2015	BCL2L2, TP53RK	Increased	26263387
miR-106b	hsa-mir-106b-5p	2015	PTEN, p21	Decreased	26238857
miR-100	hsa-mir-100-5p	2015	-	Increased	25973296
miR-622	hsa-mir-622	2015	Rb	Decreased	25961730
miR-210	hsa-mir-210-5p	2015	Bcl-2	Decreased	25385144
miR-21	hsa-mir-21-5p	2014	hMSH2	Decreased	24275137
miR-124	hsa-mir-124-3p	2014	PRRX1	Increased	24705396
miR-221	hsa-mir-221-3p	2013	PTEN	Increased	4409057

### Functional enrichment analyses

**Figure 3:**
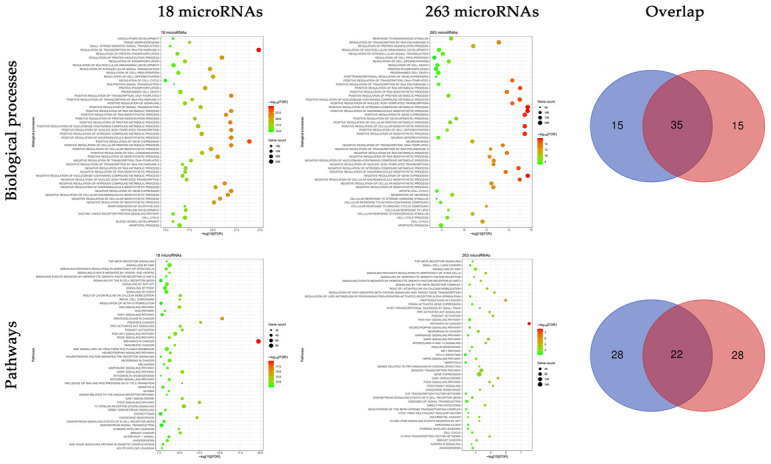
**Functional enrichment analyses of the targets of 18 experimentally validated microRNAs and 263 novel microRNAs.** Multiple biological processes and pathways were enriched in the targets of 18 experimentally validated microRNAs and 263 novel microRNAs, and there existed a high proportion of overlap.

Functional enrichment analyses were conducted on the targets of the 18 microRNAs and 263 novel microRNAs respectively. In biological processes, there was a high overlapped rate of 70% between the targets of the two groups (Figure 3). Most of these biological processes focused on the biosynthesis and of RNA and protein, and cell proliferation. In pathways, there was a relatively high overlapped rate of 44% between the targets of the two groups. Most of these pathways focused on the tumor related signaling pathways.

### Topological analysis of the sub-network of 263 microRNAs and their targets

**Figure 4:**
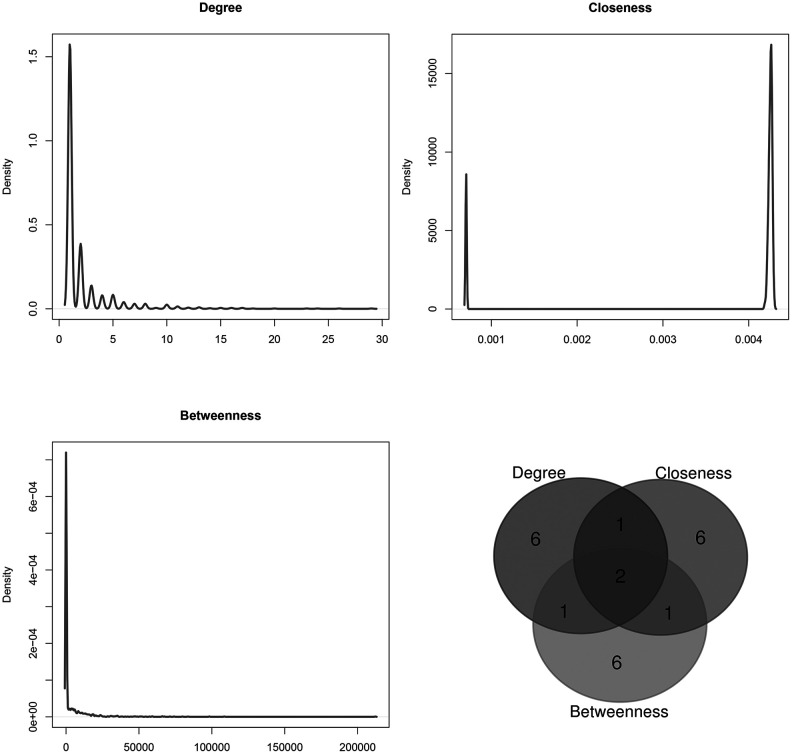
**Distribution of the sub-network topological parameters.** Topological analysis was conducted in the sub-network of 263 novel microRNAs and their targets, and the Venn diagram showed the overlap in top 10 of each dimension.

The topological features of the sub-network of 263 microRNAs and their targets were evaluated by the degree, betweenness and closeness centrality. We listed the overlap of top 10 microRNAs with topological features in each dimension (Figure 4). Two microRNAs (hsa-mir-506-3p and hsa-mir-140-5p) were identified as hub nodes in the network (Table 2).

**Table 2:** Topological features of the sub-network of 263 novel microRNAs and the targets. Two microRNAs overlapped in top 10 of each dimension.

**MicroRNA**	**Degree**	**Closeness**	**Betweenness**
Average	2.2	0.00368	3659.89585
hsa-mir-506-3p	23	0.00430	212187.38330
hsa-mir-140-5p	24	0.00429	71215.70221

### Hsa-mir-506-3p and hsa-mir-140-5p over-expression and CRCR

**Figure 5:**
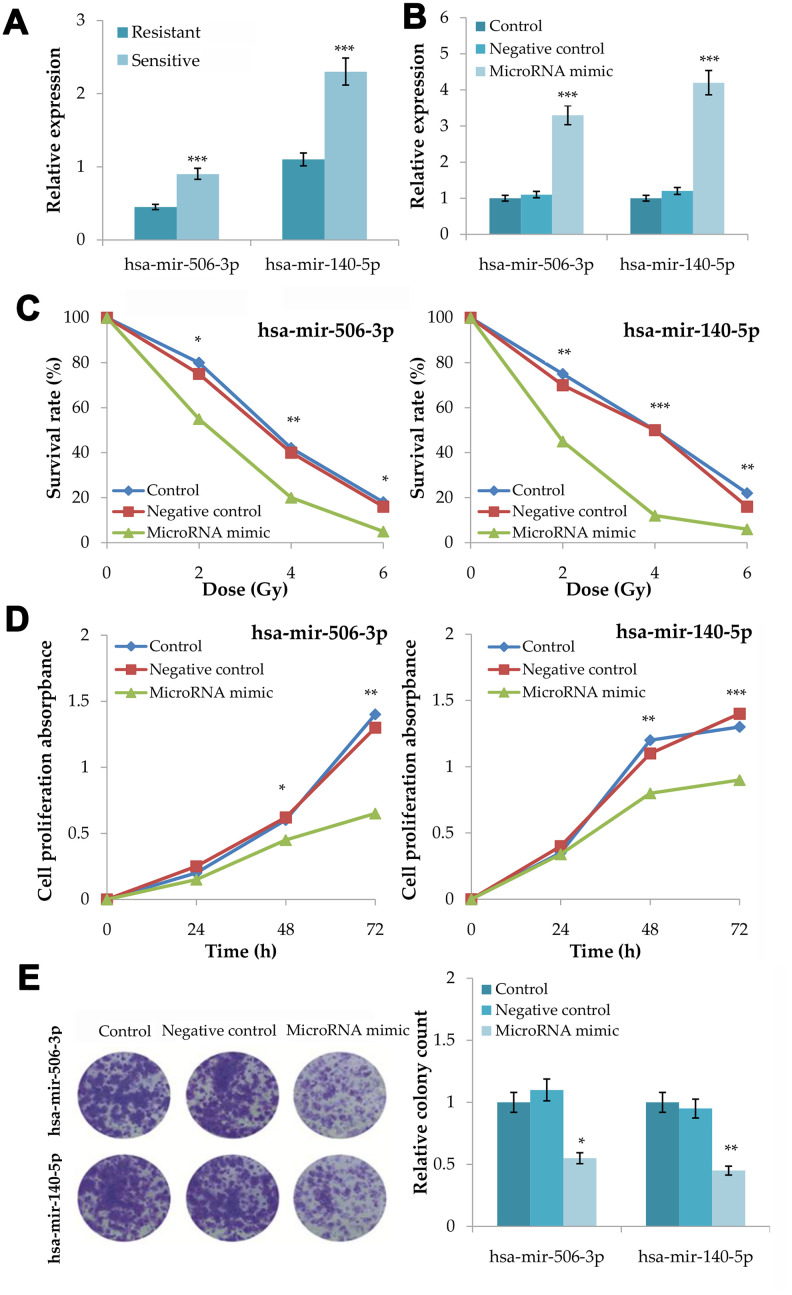
**Validation of the association between hsa-mir-506-3p and hsa-mir-140-5p and the radiosensitivity in colorectal cancer.** (**A**) Plasma expression levels of hsa-mir-506-3p and hsa-mir-140-5p between radiosensitive and radioresistant patients. (**B**) Transfection efficiency of hsa-mir-506-3p mimic and hsa-mir-140-5p mimic. (**C**) Survival curves of transfected cells with hsa-mir-506-3p mimic and hsa-mir-140-5p mimic, negative control and untransfected cells at 0, 2, 4 and 6Gy radiation. (**D**) Cell proliferation assay analysis of transfected cells with hsa-mir-506-3p mimic and hsa-mir-140-5p mimic, negative control and untransfected cells after 4Gy radiation. (**E**) Clone forming assay analysis of transfected cells with hsa-mir-506-3p mimic and hsa-mir-140-5p mimic, negative control and untransfected cells after 4Gy radiation. Compared with the control or negative control, *P<0.05, **P<0.01, ***P<0.001.

In the quantitative RT-PCR of 18 plasma samples, radiosensitive patients had a significant higher expression level of hsa-mir-506-3p (*P*=0.0004) and hsa-mir-140-5p (*P*=0.0009) than radioresistant patients (Figure 5A). Moreover, microRNA-506-3p and microRNA-140-5p showed a good diagnostic performance in CRCR (area under ROC curve (AUC) = 0.925; 0.900).

After microRNA mimic transfection into HT29 cells for 48h, the expression levels of hsa-mir-506-3p (*P*<0.001) and hsa-mir-140-5p (*P*<0.001) increased significantly (Figure 5B). After 0, 2, 4 and 6Gy radiation, the microRNA mimic group had a lower survival rate than the control group and the negative control group for hsa-mir-506-3p (*P*<0.01) and hsa-mir-140-5p (*P*<0.001) (Figure 5C). After 4Gy radiation, the microRNA mimic group had a lower growth rate than the control group and the negative control group for hsa-mir-506-3p (*P*<0.01) and hsa-mir-140-5p (*P*<0.001) (Figure 5D). After 4Gy radiation, the microRNA mimic group had a less number of colonies than the control group and the negative control group for hsa-mir-506-3p (*P*=0.0105) and hsa-mir-140-5p (*P*=0.0018) (Figure 5E).

## DISCUSSION

In this study, we built a microRNA-mRNA network based on the experimental validation computational prediction databases. Eighteen known CRCR-related microRNAs were obtained from the literature and chose as seed nodes, and then we applied the network-based RWR algorithm to identify novel CRCR-related microRNAs. Finally, 263 novel microRNAs were identified. Several microRNAs have been validated in association with the radiosensitivity in multiple cancers. For example, has-mir-339-5p could increase the radiosensitivity of lung cancer cells by targeting phosphatases of regenerating liver-1 (PRL-1) [10]. Hypoxia-responsive has-mir-301a-5p and has-mir-301b-5p promoted radioresistance of prostate cancer cells via down-regulating NDRG2 [11]. Hsa-mir-99a-5p enhanced the radiation sensitivity of non-small cell lung cancer by targeting mTOR, and inhibition of the glucocorticoid receptor resulted in an enhanced hsa-mir-99a-5p-mediated radiation response in stem-like cells from human prostate cancers [12, 13].

To validate the robustness of the prediction, we adopted two different methods. First, functional analysis was conducted respectively on the targets of the 18 microRNAs and 263 microRNAs. We found a significant overlap between the two groups, especially like the biological processes of the regulation of transcription, biosynthesis, cell proliferation and programmed cell death, as well as the pathways of MAPK signaling, PI3K-AKT signaling and TGF-β receptor signaling. These results indicated a similar role of the 263 microRNAs in the radioresistance of CRC, just like the 18 microRNAs.

To further validate our results, we also extracted the sub-network of the 263 microRNAs and their targets. After topological analysis of the sub-network, we found two hub nodes of hsa-mir-506-3p and hsa-mir-140-5p. Plasma samples of 18 CRC patients were collected to investigate the association between expression levels of the two microRNAs and subsequent radiosensitivity. Finally, we found radiosensitive patients had a significantly higher expression of both hsa-mir-506-3p and hsa-mir-140-5p than radioresistant patients. Both hsa-mir-506-3p and hsa-mir-140-5p over-expression could decrease the cell proliferation, survival rate and clonality in CRC cells after radiation. Hsa-mir-506-3p has been found in association with the progression and prognosis of non-small lung cancer and esophageal squamous cell cancer [14, 15]. Hsa-mir-140-5p could attenuate chemotherapeutic drug-induced cell death by regulating autophagy through IP3K2 in human osteosarcoma cells, and hsa-mir-140-5p could also play a therapeutic role for the treatment of non-small cell lung cancer [16, 17]. These indicated the accuracy and efficacy of our prediction. However, well-designed experiments were also needed to validate our results.

As a classical method, RWR algorithm was not as complicated as the methods in some studies mainly focusing on bioinformatics analyses. Nevertheless, we made further innovations in this study. First, we constructed the microRNA network through the bridge of microRNA-mRNA interaction data. Most of previous studies focused on the application of RWR algorithm to the mRNA network, and identified mRNA or protein biomarkers. Second, our study focused on not only the identification of novel CRC-radiosensitive microRNAs, but also the experimental validation. This combined method could enhance the reliability of bioinformatics analysis, especially in the subsequent clinical practice.

In conclusion, this study combined the novel network-based method with experimental validation, and identified two novel radiosensitive biomarkers of hsa-mir-506-3p and hsa-mir-140-5p in CRC.

## MATERIALS AND METHODS

### Random walk with restart (RWR) algorithm analysis

As a classic ranking algorithm, the RWR algorithm always simulates a random walker starting from a seed node or several seed nodes and walking on a certain network [18]. Finally, possible novel nodes are identified and ranked from high to low probabilities. The algorithm has been adopted to search novel disease genes or other related problems [19–21].

After the seed nodes are selected, the initial probability *P_0_* for each seed node is set as 1/N (where N was the number of seed nodes), while zero for non-seed nodes. Then, the RWR algorithm simulates a random walker that moved on certain network starting from seed nodes. For formulation, let *P_i_* denote a vector representing the probability of each node after the i_th_ moving procedure is complete. After each moving procedure, *P_i_* is updated as follows:

$$P_{i+1}=(1-r)\text{M}P_{i}+\text{r}P_{0}$$

where M is the column-wise normalized adjacency matrix of the network; r is the restart probability of returning to the seed nodes at every step (r was set to 0.8 in this study to indicate the importance of the known microRNAs). When the L1-norm of the difference between two successive vectors is less than 1×10^6^, the vector becomes stable, the RWR algorithm stops and output *P_i+1_* as the final vector. Each component in this vector indicates the probability of a node being trait-related. To remove the false positives, we also conducted a permutation test (1000 iterations). A false discovery rate (FDR) of less than 0.05 is considered significant.

### Identification of candidate microRNAs

In this study, we adopted the RWR algorithm in the microRNA-mRNA network to identify candidate CRCR-related microRNAs. First, we reviewed the databases of PubMed and Embase, and chose certain microRNAs as the seed nodes. The microRNAs were included if meeting the following criteria: (i) reported in association with CRCR; (ii) conducted an experimental validation; (iii) availability of the experiment details. We excluded the microRNAs without experimental validation, or only detected by bioinformatics analysis.

The microRNA-mRNA network was constructed according to the interaction data from two computational prediction databases of miRDB (http://mirdb.org/) and TargetScan (http://www.targetscan.org/), and the experimental validation database of miRTarBase (http://http://mirtarbase.cuhk.edu.cn/php/index.php). To obtain more reliable results, we only included the interactions overlapped across the databases.

Then, the sub-network of candidate microRNAs and their targets were extracted from the microRNA-mRNA network. The network degree, betweenness and closeness centrality were calculated respectively to analyze topological features of the sub-network [22]. The overlapped microRNAs with top 10 topological features in each dimension were regarded as novel CRCR biomarkers for experimental validation.

### Functional enrichment analyses

To investigate the potential function of novel microRNAs, gene ontology (GO) analyses of biological processes and pathways are performed using the online tool ToppGene (https://toppgene.cchmc.org/). A FDR of less than 0.05 is chosen as the cut-off criteria.

### Plasma samples and cell culture

The plasma samples were obtained from 18 CRC patients who subsequently underwent radiotherapy at Renmin Hospital of Wuhan University, from September to October in 2018. All patients received no other therapies before radiotherapy, and were provided written informed consent before sample collection. The present study protocol was approved by the ethics committee of Renmin Hospital of Wuhan University. The radiotherapy efficacy was classified into sensitive (n=10) and resistant (n=8) groups according to response evaluation criteria in solid tumors (RECIST).

Human CRC cell line HT29 was cultured in Dulbecco’s Modified Eagle medium (DMEM, Thermo Fisher, USA) supplemented with 10% fetal bovine serum (FBS, Thermo Fisher, USA) in a humidified incubator with 5% CO_2_ at 37°C.

### RNA extraction and quantitative RT-PCR

Total RNA was extracted using TRIzol reagent (Invitrogen, USA). The RNA concentration and purity were measured by the NanoDrop spectrophotometer (Thermo Fisher, USA). Total RNA was synthesized into first-strand cRNA using a synthesis kit (Thermo Fisher, USA). Gene expression levels were subsequently detected by the ABI Sequence Detection System (ABI, USA) using the SYBR Green Premix Ex Taq II (Takara, Japan). The relative expression levels were analyzed using the 2^−ΔΔCt^ method. Each sample was performed in triplicate. The specific primers were as follows: hsa-mir-506-3p: 5’-ACACTCATAAGGCACCCTTC-3’ (forward): 5’-TCTACTCAGAAGGGGAGTAC-3’ (reverse); hsa-mir-140-5p: 5’-CAGTGGTTTTACCCTATGGTAG-3’ (forward): 5’-ACCATAGGGTAAAACCACTGTT-3’ (reverse); GAPDH: 5’-TATAAATTGAGCCCGCAGCC-3’ (forward): 5’-TACGACCAAATCCGTTGACTC-3’ (reverse).

### MicroRNA mimic transfection

Hsa-mir-506-3p mimic, hsa-mir-140-5p mimic and their negative controls were purchased from RiboBio (Guangzhou, China). Cells were transfected with Lipofectamine 2000 (Invitrogen, USA) according to the manufacturer's protocol for 48h, and then harvested for further experiments.

### Colony forming assay

After transfection, cells were irradiated at the doses of 2, 4, and 6Gy using 250kV X-rays (Philips, the Netherlands). Then, 1000 cells were incubated in 6-well plate for 2 weeks to form visualized colonies of survived cells. Finally, cells were fixed with formaldehyde, stained with 0.1% crystal violet and air-dried. The number of clones was quantified. Plating efficiency was calculated as the ratio of the visible colonies from all seeded cells. The survival fraction was determined by the percentage of irradiated cells compared with the control.

### Cell proliferation assay

After transfection and irradiation, cells were seeded in 96-well plate with 1×10^5^/well and cultured for 24, 48 and 72h. Then, 10μl CCK8 solution (Beyotime, China) was added into each well, and the absorbance at 450nm was measured after incubating for 2h.

### Statistical analysis

All statistical analyses are performed using SPSS 20.0 (IBM, USA) and GraphPad Prism 7.0 (GraphPad, UAS). Data are presented as mean ± SD, and *t* test and one-way ANOVA are used to compare two or multiple groups. Receiver operating characteristic (ROC) curve analysis is conducted to evaluate the diagnostic ability of microRNAs. *P*<0.05 is considered statistically significant.

## Supplementary Material

Supplementary Table 1
