# Supplementary material for: RWR-algorithm-based dissection of microRNA-506-3p and microRNA-140-5p as radiosensitive biomarkers in colorectal cancer
Source: Aging (Albany NY). 2020 Oct 8;12(20):20512–22. doi: 10.18632/aging.103907 (PMC7655152; doi:10.18632/aging.103907)
Supplement: Supplementary Table 1 [file aging-12-103907-s001..docx]

**Supplementary Table 1. Novel radiosensitivity-related microRNAs identified by random walk with a restart (RWR) algorithm.**

| **microRNA** | **Probability** | **FDR** | **microRNA** | **Probability** | **FDR** | **microRNA** | **Probability** | **FDR** |
| --- | --- | --- | --- | --- | --- | --- | --- | --- |
| hsa-mir-3940-5p | 2.21E-04 | 2.00E-03 | hsa-mir-3168 | 3.18E-05 | 3.80E-02 | hsa-mir-4269 | 2.26E-05 | 3.90E-02 |
| hsa-mir-3678-5p | 2.21E-04 | 2.00E-03 | hsa-mir-2276-5p | 3.18E-05 | 3.50E-02 | hsa-mir-6715b-5p | 2.26E-05 | 3.90E-02 |
| hsa-mir-5002-3p | 1.48E-04 | 8.00E-03 | hsa-mir-6871-5p | 3.16E-05 | 1.70E-02 | hsa-mir-4471 | 2.24E-05 | 4.90E-02 |
| hsa-mir-4507 | 1.11E-04 | 4.00E-03 | hsa-mir-3663-3p | 3.16E-05 | 4.80E-02 | hsa-mir-8059 | 2.24E-05 | 4.90E-02 |
| hsa-mir-451a | 7.42E-05 | 8.00E-03 | hsa-mir-663b | 3.14E-05 | 3.80E-02 | hsa-mir-33b-5p | 2.21E-05 | 4.60E-02 |
| hsa-mir-152-5p | 7.37E-05 | 1.50E-02 | hsa-mir-4633-5p | 3.14E-05 | 2.70E-02 | hsa-mir-2861 | 2.20E-05 | 3.90E-02 |
| hsa-mir-506-3p | 7.31E-05 | 4.70E-02 | hsa-mir-7113-3p | 3.11E-05 | 4.90E-02 | hsa-mir-202-5p | 2.19E-05 | 4.30E-02 |
| hsa-mir-3649 | 7.30E-05 | 1.20E-02 | hsa-mir-3977 | 3.10E-05 | 3.50E-02 | hsa-mir-6858-3p | 2.19E-05 | 3.10E-02 |
| hsa-mir-2114-5p | 6.80E-05 | 1.40E-02 | hsa-mir-4633-3p | 3.07E-05 | 2.50E-02 | hsa-mir-6882-5p | 2.18E-05 | 3.00E-02 |
| hsa-mir-610 | 6.59E-05 | 1.70E-02 | hsa-mir-4676-5p | 3.06E-05 | 4.40E-02 | hsa-mir-4522 | 2.16E-05 | 2.30E-02 |
| hsa-mir-133a-5p | 6.42E-05 | 1.60E-02 | hsa-mir-575 | 3.06E-05 | 4.40E-02 | hsa-mir-301a-5p | 2.13E-05 | 2.50E-02 |
| hsa-mir-4462 | 6.31E-05 | 1.10E-02 | hsa-mir-1254 | 3.06E-05 | 3.20E-02 | hsa-mir-301b-5p | 2.13E-05 | 2.50E-02 |
| hsa-mir-518e-3p | 6.28E-05 | 3.60E-02 | hsa-mir-3678-3p | 3.05E-05 | 4.10E-02 | hsa-mir-624-3p | 2.13E-05 | 4.80E-02 |
| hsa-mir-668-3p | 6.27E-05 | 9.00E-03 | hsa-mir-216a-5p | 3.04E-05 | 4.70E-02 | hsa-mir-6742-3p | 2.12E-05 | 3.90E-02 |
| hsa-mir-6821-3p | 6.22E-05 | 1.30E-02 | hsa-mir-374b-3p | 3.00E-05 | 4.30E-02 | hsa-mir-8075 | 2.12E-05 | 2.40E-02 |
| hsa-mir-4317 | 6.15E-05 | 1.60E-02 | hsa-mir-582-3p | 2.98E-05 | 1.40E-02 | hsa-mir-4487 | 2.12E-05 | 4.90E-02 |
| hsa-mir-99a-5p | 6.10E-05 | 2.70E-02 | hsa-mir-488-5p | 2.96E-05 | 3.00E-02 | hsa-mir-140-5p | 2.10E-05 | 4.90E-02 |
| hsa-mir-99b-5p | 5.87E-05 | 2.00E-02 | hsa-mir-6792-5p | 2.91E-05 | 4.80E-02 | hsa-mir-4642 | 2.07E-05 | 3.50E-02 |
| hsa-mir-1295b-3p | 5.79E-05 | 1.70E-02 | hsa-mir-542-3p | 2.90E-05 | 4.60E-02 | hsa-mir-7154-5p | 2.07E-05 | 4.80E-02 |
| hsa-mir-7849-3p | 5.77E-05 | 1.60E-02 | hsa-mir-3145-5p | 2.88E-05 | 2.30E-02 | hsa-mir-4766-5p | 2.06E-05 | 4.70E-02 |
| hsa-mir-4804-3p | 5.75E-05 | 1.00E-02 | hsa-mir-27b-5p | 2.88E-05 | 3.10E-02 | hsa-mir-5586-5p | 2.04E-05 | 4.60E-02 |
| hsa-mir-4687-5p | 5.56E-05 | 1.20E-02 | hsa-mir-4448 | 2.86E-05 | 3.30E-02 | hsa-mir-3162-3p | 2.04E-05 | 4.40E-02 |
| hsa-mir-7108-3p | 5.54E-05 | 1.60E-02 | hsa-mir-623 | 2.86E-05 | 4.40E-02 | hsa-mir-6831-3p | 2.03E-05 | 3.10E-02 |
| hsa-mir-3622a-5p | 5.53E-05 | 1.50E-02 | hsa-mir-4501 | 2.85E-05 | 2.80E-02 | hsa-mir-4521 | 2.03E-05 | 3.00E-02 |
| hsa-mir-1292-5p | 5.52E-05 | 3.50E-02 | hsa-mir-3661 | 2.84E-05 | 3.60E-02 | hsa-mir-4511 | 2.02E-05 | 4.90E-02 |
| hsa-mir-8056 | 5.50E-05 | 1.30E-02 | hsa-mir-631 | 2.84E-05 | 3.60E-02 | hsa-mir-193a-3p | 2.00E-05 | 3.60E-02 |
| hsa-mir-500b-5p | 4.92E-05 | 3.80E-02 | hsa-mir-6764-3p | 2.84E-05 | 3.80E-02 | hsa-mir-769-5p | 2.00E-05 | 3.90E-02 |
| hsa-mir-4327 | 4.91E-05 | 1.50E-02 | hsa-mir-148a-5p | 2.82E-05 | 3.70E-02 | hsa-mir-197-3p | 1.94E-05 | 3.80E-02 |
| hsa-mir-6503-3p | 4.45E-05 | 1.30E-02 | hsa-mir-4299 | 2.81E-05 | 3.70E-02 | hsa-mir-564 | 1.93E-05 | 3.90E-02 |
| hsa-mir-145-3p | 4.43E-05 | 2.10E-02 | hsa-mir-7158-5p | 2.80E-05 | 3.80E-02 | hsa-mir-7152-5p | 1.91E-05 | 4.60E-02 |
| hsa-mir-6794-3p | 4.42E-05 | 3.30E-02 | hsa-mir-592 | 2.79E-05 | 3.60E-02 | hsa-mir-1293 | 1.89E-05 | 4.50E-02 |
| hsa-mir-4751 | 4.40E-05 | 3.10E-02 | hsa-mir-371a-3p | 2.79E-05 | 3.00E-02 | hsa-mir-4445-5p | 1.88E-05 | 3.50E-02 |
| hsa-mir-3911 | 4.36E-05 | 1.90E-02 | hsa-mir-7156-3p | 2.78E-05 | 3.90E-02 | hsa-mir-4300 | 1.88E-05 | 3.80E-02 |
| hsa-mir-3664-5p | 4.36E-05 | 2.40E-02 | hsa-mir-7151-5p | 2.78E-05 | 3.80E-02 | hsa-mir-6726-5p | 1.88E-05 | 3.80E-02 |
| hsa-mir-3161 | 4.36E-05 | 2.00E-02 | hsa-let-7i-3p | 2.78E-05 | 2.40E-02 | hsa-mir-920 | 1.88E-05 | 3.80E-02 |
| hsa-mir-1193 | 4.30E-05 | 2.70E-02 | hsa-mir-6772-5p | 2.78E-05 | 2.40E-02 | hsa-mir-188-5p | 1.84E-05 | 4.50E-02 |
| hsa-mir-2681-3p | 4.28E-05 | 3.60E-02 | hsa-mir-137 | 2.78E-05 | 3.60E-02 | hsa-mir-3689a-5p | 1.81E-05 | 3.40E-02 |
| hsa-mir-3976 | 4.17E-05 | 2.70E-02 | hsa-mir-432-3p | 2.77E-05 | 2.50E-02 | hsa-mir-3689b-5p | 1.81E-05 | 3.40E-02 |
| hsa-mir-4438 | 4.08E-05 | 3.40E-02 | hsa-mir-6736-3p | 2.77E-05 | 3.50E-02 | hsa-mir-3689e | 1.81E-05 | 3.40E-02 |
| hsa-mir-668-5p | 4.04E-05 | 2.50E-02 | hsa-mir-605-5p | 2.76E-05 | 4.10E-02 | hsa-mir-3689f | 1.81E-05 | 3.40E-02 |
| hsa-mir-135b-5p | 4.03E-05 | 2.50E-02 | hsa-mir-6500-5p | 2.76E-05 | 1.70E-02 | hsa-mir-1286 | 1.79E-05 | 4.00E-02 |
| hsa-mir-490-5p | 3.95E-05 | 1.30E-02 | hsa-mir-4802-5p | 2.75E-05 | 4.20E-02 | hsa-mir-652-3p | 1.79E-05 | 4.20E-02 |
| hsa-mir-4490 | 3.94E-05 | 3.30E-02 | hsa-mir-214-5p | 2.75E-05 | 2.20E-02 | hsa-mir-455-5p | 1.78E-05 | 4.20E-02 |
| hsa-mir-191-5p | 3.89E-05 | 2.30E-02 | hsa-mir-6772-3p | 2.74E-05 | 3.30E-02 | hsa-mir-6857-5p | 1.78E-05 | 4.50E-02 |
| hsa-mir-532-3p | 3.83E-05 | 2.80E-02 | hsa-mir-609 | 2.72E-05 | 3.90E-02 | hsa-mir-1261 | 1.76E-05 | 4.30E-02 |
| hsa-mir-590-5p | 3.82E-05 | 3.20E-02 | hsa-mir-6801-5p | 2.72E-05 | 2.00E-02 | hsa-mir-8082 | 1.74E-05 | 4.80E-02 |
| hsa-mir-6824-3p | 3.76E-05 | 2.10E-02 | hsa-mir-320c | 2.71E-05 | 3.30E-02 | hsa-mir-5684 | 1.74E-05 | 4.20E-02 |
| hsa-mir-337-3p | 3.74E-05 | 3.10E-02 | hsa-mir-6761-5p | 2.68E-05 | 3.30E-02 | hsa-mir-892b | 1.73E-05 | 4.30E-02 |
| hsa-mir-320e | 3.73E-05 | 2.40E-02 | hsa-mir-4703-5p | 2.67E-05 | 2.10E-02 | hsa-mir-4796-5p | 1.73E-05 | 4.90E-02 |
| hsa-mir-3155a | 3.72E-05 | 2.10E-02 | hsa-mir-4774-3p | 2.65E-05 | 2.00E-02 | hsa-mir-6866-5p | 1.72E-05 | 4.70E-02 |
| hsa-mir-6508-3p | 3.70E-05 | 2.20E-02 | hsa-mir-379-5p | 2.64E-05 | 3.00E-02 | hsa-mir-4728-3p | 1.70E-05 | 4.80E-02 |
| hsa-mir-6818-3p | 3.69E-05 | 4.60E-02 | hsa-mir-193a-5p | 2.62E-05 | 3.60E-02 | hsa-mir-6859-3p | 1.68E-05 | 2.40E-02 |
| hsa-mir-1322 | 3.69E-05 | 2.20E-02 | hsa-mir-3189-3p | 2.62E-05 | 3.50E-02 | hsa-mir-4691-5p | 1.67E-05 | 3.70E-02 |
| hsa-mir-7112-5p | 3.69E-05 | 4.30E-02 | hsa-mir-632 | 2.61E-05 | 2.40E-02 | hsa-mir-514a-5p | 1.66E-05 | 3.60E-02 |
| hsa-mir-4732-5p | 3.68E-05 | 3.80E-02 | hsa-mir-711 | 2.55E-05 | 3.30E-02 | hsa-mir-6861-3p | 1.65E-05 | 4.60E-02 |
| hsa-mir-338-3p | 3.68E-05 | 3.80E-02 | hsa-mir-6833-5p | 2.54E-05 | 4.30E-02 | hsa-mir-29b-1-5p | 1.65E-05 | 3.80E-02 |
| hsa-mir-3117-5p | 3.64E-05 | 2.50E-02 | hsa-mir-627-5p | 2.54E-05 | 3.60E-02 | hsa-mir-4750-3p | 1.61E-05 | 3.40E-02 |
| hsa-mir-3164 | 3.63E-05 | 4.60E-02 | hsa-mir-4433a-3p | 2.51E-05 | 4.60E-02 | hsa-mir-6804-3p | 1.59E-05 | 3.30E-02 |
| hsa-mir-5007-5p | 3.62E-05 | 2.60E-02 | hsa-mir-6730-3p | 2.50E-05 | 2.90E-02 | hsa-mir-1206 | 1.56E-05 | 3.40E-02 |
| hsa-mir-3184-3p | 3.55E-05 | 4.40E-02 | hsa-mir-4727-3p | 2.50E-05 | 2.40E-02 | hsa-mir-936 | 1.51E-05 | 4.00E-02 |
| hsa-mir-3180-5p | 3.51E-05 | 4.20E-02 | hsa-mir-5195-5p | 2.48E-05 | 2.40E-02 | hsa-mir-875-5p | 1.49E-05 | 3.90E-02 |
| hsa-mir-4423-5p | 3.51E-05 | 3.10E-02 | hsa-mir-3165 | 2.48E-05 | 2.90E-02 | hsa-mir-1263 | 1.49E-05 | 3.30E-02 |
| hsa-mir-1265 | 3.44E-05 | 3.50E-02 | hsa-mir-8070 | 2.48E-05 | 3.80E-02 | hsa-mir-7159-3p | 1.41E-05 | 4.10E-02 |
| hsa-mir-136-5p | 3.43E-05 | 2.50E-02 | hsa-mir-1255a | 2.48E-05 | 2.80E-02 | hsa-mir-3682-3p | 1.41E-05 | 3.70E-02 |
| hsa-mir-103a-2-5p | 3.41E-05 | 3.40E-02 | hsa-mir-1255b-5p | 2.48E-05 | 2.80E-02 | hsa-mir-515-3p | 1.41E-05 | 4.70E-02 |
| hsa-mir-4782-5p | 3.39E-05 | 3.20E-02 | hsa-mir-491-3p | 2.46E-05 | 2.80E-02 | hsa-mir-519e-3p | 1.41E-05 | 4.70E-02 |
| hsa-mir-5706 | 3.39E-05 | 3.20E-02 | hsa-mir-567 | 2.45E-05 | 4.40E-02 | hsa-mir-1273a | 1.41E-05 | 3.30E-02 |
| hsa-mir-4655-5p | 3.39E-05 | 2.10E-02 | hsa-mir-6888-5p | 2.45E-05 | 3.60E-02 | hsa-mir-6877-5p | 1.39E-05 | 4.90E-02 |
| hsa-mir-6510-5p | 3.37E-05 | 3.40E-02 | hsa-mir-758-3p | 2.44E-05 | 4.50E-02 | hsa-mir-6884-3p | 1.39E-05 | 4.50E-02 |
| hsa-mir-6874-5p | 3.36E-05 | 2.80E-02 | hsa-mir-483-3p | 2.43E-05 | 2.70E-02 | hsa-mir-4709-5p | 1.38E-05 | 4.10E-02 |
| hsa-mir-6830-5p | 3.34E-05 | 2.60E-02 | hsa-mir-1266-3p | 2.43E-05 | 3.30E-02 | hsa-mir-15b-3p | 1.38E-05 | 3.80E-02 |
| hsa-mir-339-5p | 3.33E-05 | 2.20E-02 | hsa-mir-518d-5p | 2.43E-05 | 4.50E-02 | hsa-mir-190b | 1.37E-05 | 4.40E-02 |
| hsa-mir-3659 | 3.32E-05 | 2.90E-02 | hsa-mir-299-5p | 2.41E-05 | 4.90E-02 | hsa-mir-574-3p | 1.36E-05 | 3.90E-02 |
| hsa-mir-6769a-3p | 3.31E-05 | 2.90E-02 | hsa-mir-5588-3p | 2.40E-05 | 2.80E-02 | hsa-mir-503-3p | 1.36E-05 | 4.00E-02 |
| hsa-mir-3188 | 3.29E-05 | 4.20E-02 | hsa-mir-1276 | 2.40E-05 | 4.80E-02 | hsa-mir-4718 | 1.33E-05 | 4.90E-02 |
| hsa-mir-218-1-3p | 3.29E-05 | 2.00E-02 | hsa-mir-4474-3p | 2.39E-05 | 2.60E-02 | hsa-mir-5591-5p | 1.27E-05 | 4.00E-02 |
| hsa-mir-1178-3p | 3.29E-05 | 3.90E-02 | hsa-mir-6828-3p | 2.39E-05 | 4.10E-02 | hsa-mir-5571-3p | 1.25E-05 | 4.50E-02 |
| hsa-mir-1226-5p | 3.29E-05 | 4.00E-02 | hsa-mir-6792-3p | 2.38E-05 | 3.30E-02 | hsa-mir-6729-3p | 1.24E-05 | 4.60E-02 |
| hsa-mir-1245b-5p | 3.28E-05 | 3.00E-02 | hsa-mir-374c-5p | 2.38E-05 | 4.30E-02 | hsa-mir-3116 | 1.22E-05 | 4.90E-02 |
| hsa-mir-6881-3p | 3.27E-05 | 4.40E-02 | hsa-mir-3688-5p | 2.38E-05 | 2.50E-02 | hsa-mir-154-3p | 1.19E-05 | 4.10E-02 |
| hsa-mir-5191 | 3.26E-05 | 4.20E-02 | hsa-mir-892c-3p | 2.38E-05 | 2.80E-02 | hsa-mir-487a-3p | 1.19E-05 | 4.10E-02 |
| hsa-mir-591 | 3.25E-05 | 3.70E-02 | hsa-mir-6501-3p | 2.37E-05 | 4.80E-02 | hsa-mir-589-5p | 1.17E-05 | 4.80E-02 |
| hsa-mir-371b-3p | 3.24E-05 | 2.90E-02 | hsa-mir-146b-5p | 2.37E-05 | 4.80E-02 | hsa-mir-3677-5p | 1.04E-05 | 4.10E-02 |
| hsa-mir-6872-3p | 3.24E-05 | 3.30E-02 | hsa-mir-508-3p | 2.33E-05 | 3.70E-02 | hsa-mir-3614-3p | 1.03E-05 | 3.10E-02 |
| hsa-mir-151a-5p | 3.22E-05 | 3.40E-02 | hsa-mir-5087 | 2.31E-05 | 4.80E-02 | hsa-mir-1238-5p | 1.03E-05 | 4.00E-02 |
| hsa-mir-8080 | 3.20E-05 | 4.40E-02 | hsa-mir-7856-5p | 2.30E-05 | 3.00E-02 | hsa-mir-4758-5p | 1.03E-05 | 4.00E-02 |
| hsa-mir-150-3p | 3.19E-05 | 4.10E-02 | hsa-mir-5581-3p | 2.28E-05 | 4.60E-02 | hsa-mir-571 | 1.01E-05 | 3.40E-02 |
| hsa-mir-3137 | 3.19E-05 | 2.20E-02 | hsa-mir-548b-3p | 2.26E-05 | 3.60E-02 |  |  |  |
